# Deep learning-based classification of nitrate and nitrite concentrations from water samples using colorimetric test strip images

**DOI:** 10.1007/s10661-026-15297-y

**Published:** 2026-04-11

**Authors:** Muhammad Roman, Mazhar Sher, Chamika Kuruppuarachchi, Arshid Ali, Chulwoo Pack, Azlan Zahid, Ali Mirzakhani Nafchi

**Affiliations:** 1 Department of Agricultural and Biosystems Engineering, South Dakota State University, Brookings, SD 57007 USA; 2 Department of Electrical Engineering and Computer Science, South Dakota State University, Brookings, SD 57007 USA; 3 Department of Biological and Agricultural Engineering, Texas A&M AgriLife Research, Texas A&M University System, Dallas, TX 75252 USA; 4 Department of Agronomy, Horticulture and Plant Science, College of Agriculture, Food and Environmental Sciences, South Dakota State University, Brookings, SD 57007 USA

**Keywords:** Precision agriculture, Image classification, Water quality monitoring, Deep learning

## Abstract

Accurate monitoring of nitrate and nitrite concentrations in water is essential for sustainable agriculture, safeguarding public health, and protecting aquatic ecosystems from nutrient pollution. Traditional methods for detecting nitrate and nitrite in water samples are precise but costly, complex, and time-consuming, limiting their practicality for frequent on-site testing. This research proposes deep learning-based computer vision techniques to classify nitrate and nitrite concentrations using images of colorimetric test strips. An RGB IMX219 camera was used to acquire images of colorimetric test strips under standardized, controlled illumination conditions to ensure consistent image quality. A total of 1938 nitrate images and 1190 nitrite images were collected before augmentation. After preprocessing and training-only data augmentation, both classical machine learning baselines based on hand-crafted color and texture features and deep learning models—including a multilayer perceptron (MLP) and convolutional neural networks (AlexNet, VGG16, ResNet18, and GoogLeNet)—were trained and evaluated using an independent test set and stratified fivefold cross-validation. For nitrate classification, ResNet18 and GoogLeNet achieved near-perfect 100% test accuracy, with mean cross-validation accuracy of 99.97% ± 0.04%, substantially outperforming classical baseline models based on hand-crafted color and texture features, which achieved at most 83.5% test accuracy. For nitrite classification, GoogLeNet achieved the strongest overall performance, with a test accuracy of 97.48% and a fivefold cross-validation accuracy of 95.22% ± 1.17%, substantially outperforming the best classical baseline model, which achieved a maximum test accuracy of 83.19%. These results demonstrate that deep CNN-based feature learning provides a significant performance advantage over simpler methods under controlled imaging conditions, supporting the suitability of the proposed system for rapid, image-based water quality assessment and motivating future evaluation under broader real-world deployment scenarios.

## Introduction

Water is a fundamental natural resource essential for the sustenance and survival of human life (Alizamir et al., 2025). In recent years, the accelerating pace of industrialization, urban development, and intensive agricultural activities has affected the water quality on a global scale (Akhtar et al., 2021; Saxena, 2025). Freshwater is increasingly threatened by human-caused pollutants, including chemical waste, heavy metals, pathogens, and organic matter, which tend to be discharged into river systems, lakes, and groundwater reservoirs without having been treated (Mishra, 2023; Mushtaq et al., 2020). These contaminants also pose a great threat to the environment and public health, particularly in regions with limited sources of clean water infrastructure. As a result, ensuring access to clean water is now one of the most urgent global health and environmental challenges of the twenty-first century (Elimelech, 2006; Levin et al., 2002). Therefore, the prediction and assessment of water quality constitute a critical task for ensuring environmental sustainability and safeguarding public health (Yan et al., 2022).

Nitrate and nitrite are among the most critical chemical contaminants affecting water quality (Kensington et al., 2004). The primary sources of nitrate and nitrite contamination in water include agricultural runoff from excessive fertilizer use, as well as contributions from industrial waste, septic systems, and livestock manure. These compounds are leached into groundwater and are transported through surface water, due to their high solubility, thus making them prevalent in both rural and urban water supplies (Craswell, 2021). Prolonged exposure to these chemical compounds in drinking water can have serious health consequences, such as methemoglobinemia (known as “blue baby syndrome”) (Majumdar, 2003), increased risk of certain cancers (Picetti et al., 2022), and disruptions to the human endocrine and cardiovascular systems (Bryan & Loscalzo, 2017). These pollutants contribute to eutrophication, resulting in oxygen depletion and loss of aquatic biodiversity (Jan et al., 2022). Due to their impact on human health and the environment, continuous water quality monitoring is critical for regulatory compliance, public safety, and ecosystem protection.

Standard methods for measuring nitrate and nitrite concentrations in water, such as ion chromatography, ultraviolet–visible (UV–Vis) spectrophotometry, and electrochemical methods, are recognized for their high quality and reliability in laboratory settings. Ion chromatography (IC) is a widely used technique for accurately separating and quantifying ions in water samples; however, it requires expensive instrumentation and skilled operation (Connolly & Paull, 2001). Spectrophotometry relies on the absorption characteristics of nitrate and nitrite compounds at specific wavelengths, providing reliable results but often involving complex reagents and sample preparation steps (Narayana & Sunil, 2009). Ion-selective electrodes and voltammetric techniques offer direct detection with reasonable sensitivity but are prone to interference from co-existing ions and often require frequent calibration (Revsbech et al., 2020). Despite their analytical accuracy, these methods are limited by high cost, complex sample preparation, dependence on laboratory infrastructure, and delayed processing times, which restrict their suitability for large-scale, real-time monitoring. Therefore, there is a clear need for rapid, affordable, and user-friendly methods for measuring nitrate and nitrite concentrations with high reliability and accuracy.

Recent advances in artificial intelligence (AI), particularly in computer vision and deep learning, have opened new areas for efficient and automated water quality monitoring (Gunda et al., 2019; Ighalo et al., 2021; Yang et al., 2022). These techniques enable effective extraction of spatial features from visual data, significantly improving contaminant detection performance. Compared to traditional machine learning models, convolutional neural networks (CNNs) have demonstrated superior performance due to their ability to automatically learn hierarchical representations and effectively generalize across varied datasets (Esteva et al., 2017; LeCun et al., 2015). Specifically, GoogLeNet, ResNet18, and VGG16 models have been widely chosen for environmental and agricultural monitoring tasks because of their demonstrated robustness, effectiveness in feature extraction, and high classification accuracy (Wu et al., 2025; Yang et al., 2021; Yuesheng et al., 2021). Unlike traditional methods, AI-based systems can be deployed in low-cost, portable setups, enabling rapid on-site assessments with minimal human intervention.

Vision-based analysis of colorimetric test strips has emerged as a promising alternative for nitrate measurement using camera or smartphone-assisted approaches. However, many existing methods rely on manual RGB feature extraction, calibration curves, and uncontrolled imaging conditions, which can limit robustness and repeatability. Prior studies have reported that smartphone-based quantification of nitrate test strips does not consistently outperform visual interpretation, highlighting the influence of imaging conditions and user-dependent variability (Topping & Kolok, 2021). Moreover, vision-based systems targeting fine-grained, multi-class classification of nitrate and nitrite concentrations remain limited. These challenges motivate the need for learning-based approaches that can automatically capture subtle spatial and color patterns under standardized imaging conditions.

In this study, we propose a computer vision-based pipeline that leverages both classical and deep learning models to detect and classify nitrate and nitrite concentrations from the water test strip images. Classical baseline models, including logistic regression (LR), linear support vector machine (SVM), and Naïve Bayes (NB), are incorporated to provide interpretable reference performance and to quantify the benefits of deep feature learning. In parallel, several deep learning architectures are evaluated, including a multilayer perceptron (MLP) and several convolutional neural networks (CNNs) such as AlexNet (Pu et al., 2019), VGG16 (Al-Sudani & Al-Suhail, 2024), ResNet18 (Malathi et al., 2023), and GoogLeNet (Sumaya et al., 2024), each selected for their proven effectiveness in image classification tasks. Using a standardized image dataset, we evaluated each model’s learning capability, classification accuracy, and computational efficiency to assess their suitability for real-time, scalable deployment in water quality monitoring applications. Key contributions include the application of multiple deep learning models for multi-class classification of nitrate and nitrite concentrations and a comprehensive performance analysis to identify the most effective models for future integration into AI-powered water monitoring systems.

## Methodology

**Fig. 1:**
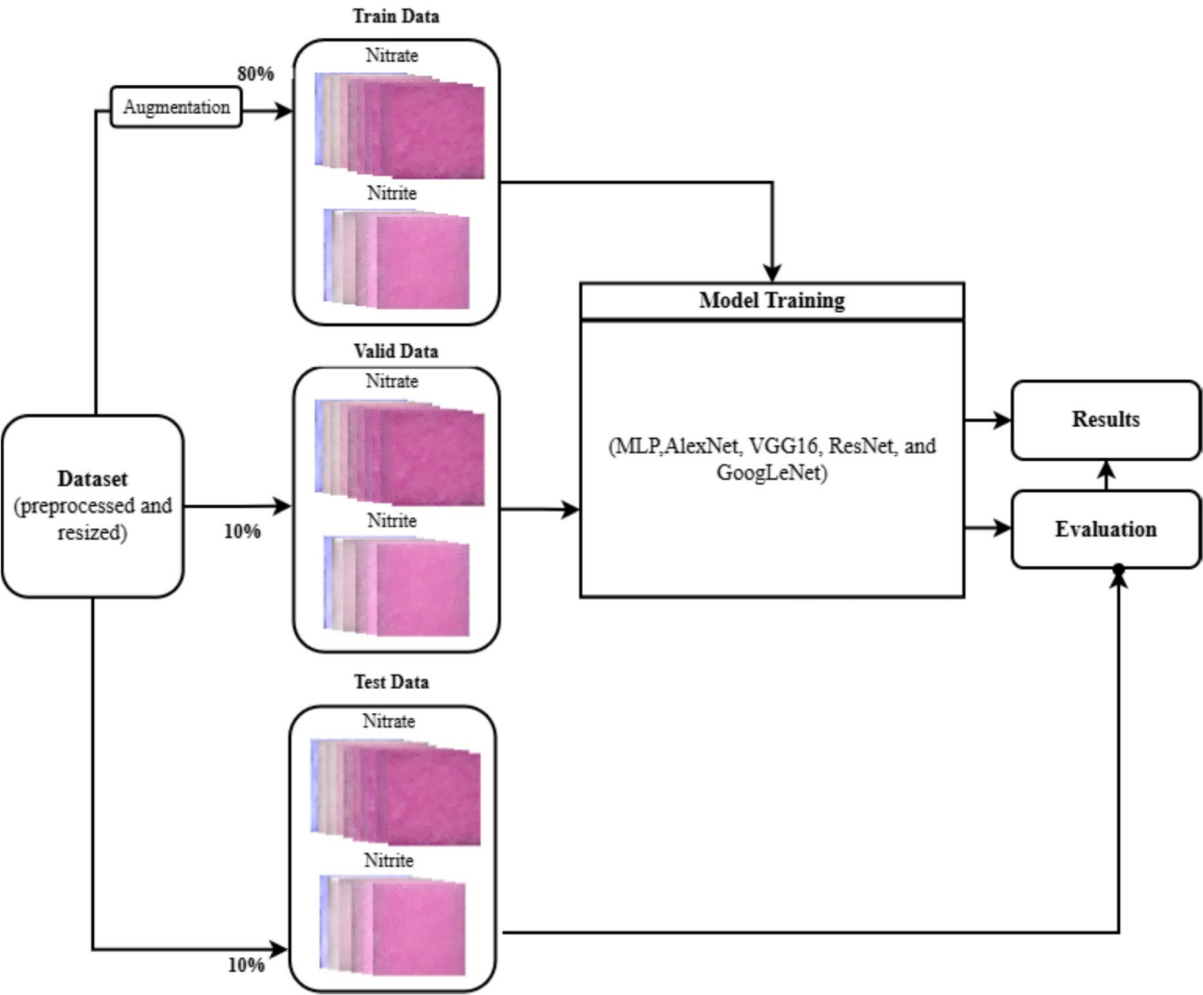
System Architecture flow diagram of nitrate and nitrite classification

The overall architecture of the proposed system comprises a streamlined pipeline for classifying nitrate and nitrite concentrations from test strip dataset images using deep learning techniques. The system is composed of three main stages: dataset preparation, model training, and evaluation. In the first stage, the labeled dataset of nitrate and nitrite is prepared and divided into 80:10:10 ratios for training, validation, and testing. The dataset was preprocessed and resized to a standard size. The trained dataset was augmented. In the second step, latent features were extracted from the training dataset using five deep learning models. Model training and validation were performed using Google Colab, leveraging GPU acceleration to reduce computation time and optimize model performance. Each model received the same input data to ensure a fair performance comparison. Finally, in the evaluation stage, the models were assessed using evaluation matrices to visualize their performance across different classes. The overall system architecture is shown in Fig. 1.

### Dataset

Commercially available Hach water quality test strips for nitrate and nitrite were used to collect the image dataset. The test strips were immersed in the water samples with known concentrations. After color development, the test strip image was captured with an IMX219 camera inside a custom-designed, 3D-printed, enclosed imaging box under controlled lighting. The enclosed setup ensured a fixed camera position and illumination geometry, minimizing the influence of ambient lighting, shadows, and background variations during image acquisition. Each image is stored according to the corresponding nitrate and nitrite concentration, resulting in a well-structured dataset suitable for deep learning applications. The specific concentrations used for sample preparation are detailed in Table 1. To enhance computational efficiency, the images were preprocessed to reduce noise using a median filter and uniformly resized to 64 × 64 pixels. This dataset was created by the Biosensors lab SDSU (Muhammad Roman et al., 2026) .

**Table 1:** Nitrate and nitrite sample concentrations

Nitrate samples (PPM)	Nitrite samples (PPM)
0, 1, 2, 5, 10, 15, 20, 30, 40, 50	0.15, 0.3, 1.0, 1.5, 3.0

#### a. Nitrate-nitrite dataset

**Fig. 2:**
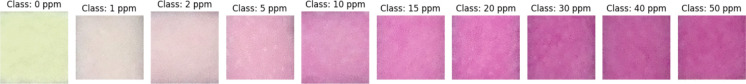
Random 1 sample from each nitrate class

**Fig. 3:**
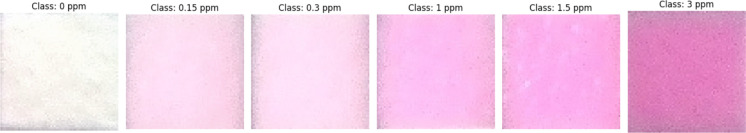
Random 1 sample from each nitrite class

The initial image datasets for both nitrate and nitrite classification contained approximately 200 images per class. Each image was captured using a new test strip with a known concentration of nitrate and nitrite. The dataset was divided into training, validation, and test sets using an 80/10/10 split on a per-class basis to preserve class balance across all concentrations. Data augmentation was applied only to the training set, while validation and test sets remained unchanged to ensure unbiased performance evaluation. Augmentation techniques included random rotations (± 90°), horizontal flipping, and controlled brightness variation (± 10%) to simulate minor illumination changes, while the imaging system itself operated in an enclosed environment to minimize external lighting effects. The distribution of training and testing images per class, along with total counts, is presented in Table 2 for nitrate and Table 3 for nitrite. Representative sample images from each class are shown in Figs. 2 and 3, respectively.

**Table 2:** Nitrate dataset images before and after augmentation

	Class (ppm)	Train (before Aug)	Train (after Aug)	Val	Test	Total
Nitrate	0	94	376	12	12	400
	1	180	720	23	23	766
	2	171	684	22	22	728
	5	153	612	19	19	650
	10	177	708	22	22	752
	15	137	548	17	17	582
	20	163	652	20	20	692
	30	155	620	19	19	658
	40	156	624	19	19	662
	50	164	656	21	21	698
	**Total**	**1550**	**6200**	**194**	**194**	**6588**

**Table 3:** Nitrite dataset images before and after augmentation

	Class (ppm)	Train (before Aug)	Train (after Aug)	Val	Test	Total
**Nitrite**	0	132	528	16	16	560
	0.15	167	668	21	21	710
	0.3	168	672	21	21	714
	1	166	664	21	21	706
	1.5	164	656	20	20	696
	3	155	620	20	20	660
	**Total**	**952**	**3808**	**119**	**119**	**4046**

### Deep learning architecture

This study utilizes several deep learning models to classify nitrate and nitrite concentrations from colorimetric strip images. The selected models include traditional architectures (MLP, AlexNet, VGG16) and more advanced designs (ResNet18, GoogLeNet), enabling a comparative analysis of performance across different levels of complexity and feature learning capabilities.

#### a) Multilayer perceptron

**Fig. 4:**
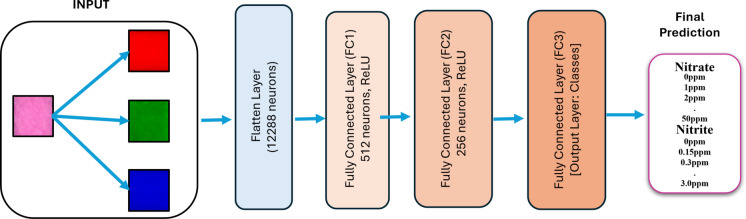
Multilayer perceptron network model architecture

The Multilayer Perceptron is a feedforward neural network composed of dense, fully connected layers, as shown in Fig. 4. It accepts vectorized input and relies on non-linear activations to learn feature representations. MLPs are often used as baselines due to their simplicity and fast training, but they lack spatial context awareness inherent to image data (Foody, 2004).

#### b) AlexNet

**Fig. 5:**
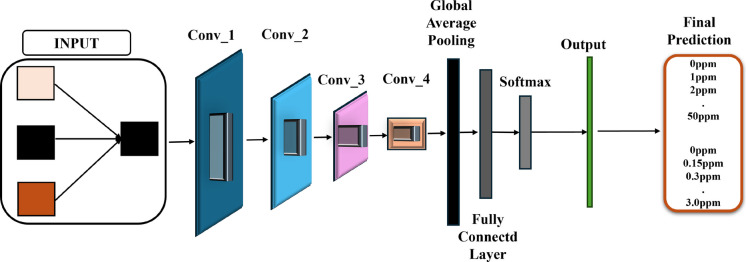
AlexNet model architecture

AlexNet is one of the first deep CNNs to demonstrate the potential of deep learning for image recognition tasks. The network architecture (Lorenzo et al., 2020) is shown in Fig. 5. It consists of five convolutional layers followed by three fully connected layers and introduces the use of ReLU activations and dropout regularization. Although pioneering, AlexNet’s architecture includes large filters and pooling steps that can limit its performance on low-resolution images (Chen et al., 2022).

#### c) VGG16

**Fig. 6:**
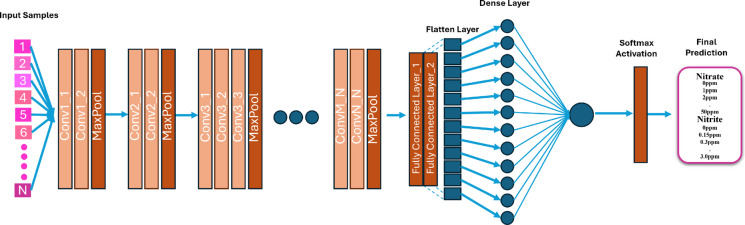
VGG16 network architecture

VGG16 is a deep CNN architecture that uses a stack of small 3 × 3 convolutional filters across 16 weight layers. Its uniform and simple structure has made it widely adopted for visual recognition benchmarks. However, the large number of parameters in VGG16 can lead to high memory consumption and overfitting when used on smaller datasets (Simonyan & Zisserman, 2014). The network architecture (Shazia et al., 2021) is shown in Fig. 6.

#### d) ResNet18

**Fig. 7:**
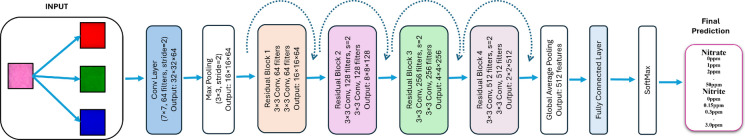
ResNet18 model architecture

ResNet18 introduced the concept of residual learning by incorporating skip connections that bypass one or more layers. These identity mappings improve gradient flow during backpropagation, enabling the training of deeper networks with fewer convergence issues. ResNet18 is a widely used balanced architecture known for its efficiency and strong generalization (He et al., 2016). Figure 7 highlights the core components of the ResNet18 Network (MohammadiNasab, 2023).

#### e) GoogLeNet

**Fig. 8:**
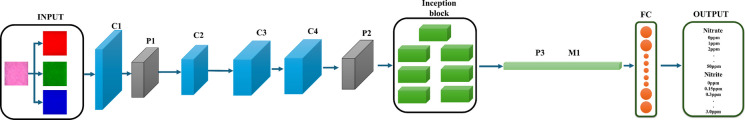
Block diagram of the GoogLeNet architecture with inception modules used for classification

GoogLeNet is a CNN architecture that utilizes inception modules, combinations of 1 × 1, 3 × 3, and 5 × 5 convolutions executed in parallel, to efficiently capture multiscale spatial information. The inclusion of 1 × 1 convolutions reduces dimensionality, making the network both deep and computationally efficient. It also incorporates auxiliary classifiers to stabilize training and reduce vanishing gradients (Abd El-Maksoud et al., 2021). GoogLeNet system architecture (Alkan et al., 2021) is shown in Fig. 8.

### Classical machine learning baseline models

In addition to deep learning architectures, classical machine learning models were implemented to provide baseline performance comparisons and to quantify the benefit of deep feature learning. These models rely on hand-crafted image features rather than end-to-end representation learning and are commonly used in traditional colorimetric and image-based analysis tasks.

Three simple yet representative feature sets were extracted from the colorimetric strip images: mean HSV color features to capture global color intensity, hue histograms to represent color distribution variations across concentrations, and local binary pattern (LBP) histograms to describe basic texture information within the reaction regions. Using these features, three baseline classifiers were evaluated: logistic regression (Arief Subchan & Andayani, 2021), linear support vector machine (SVM) (Ebrahimi et al., 2017), and Gaussian Naïve Bayes (Agarwal et al., 2018).

All baseline models were trained and evaluated using the same train/validation/test splits as the deep learning models to ensure a fair comparison. These classical baselines provide interpretable reference models and highlight the limitations of hand-crafted features when compared to deep convolutional neural networks.

### Training

Each model was trained using a categorical cross-entropy loss function optimized with the Adam optimizer. The initial learning rate was set to 0.001 for MLP and ResNet18, and 0.0005 for AlexNet, VGG16, and GoogLeNet, with a batch size of 32. Training was conducted for up to 50 epochs, with early stopping based on validation loss to prevent overfitting.

To ensure stable convergence, a StepLR learning rate scheduler was employed with a decay factor of 0.1 applied at fixed intervals during training. Model performance was monitored using a dedicated validation set, while the test set was used only for final evaluation. All experiments were conducted using fixed random seeds and deterministic training settings to ensure reproducibility.

The MLP model consisted of fully connected layers with ReLU activation functions. Due to its straightforward structure, the MLP served as a baseline for comparative performance analysis. AlexNet was trained with its original architecture of five convolutional layers followed by three fully connected layers. Dropout regularization was applied after the fully connected layers to mitigate overfitting. The VGG16 model fine-tunes its original deep convolutional architecture. Fine-tuning involved training the last convolutional block along with fully connected layers to adapt the model specifically to the nitrate and nitrite image dataset. The ResNet18 model leveraged residual connections to maintain effective gradient flow during training. Fine-tuning was performed by training all residual blocks with emphasis on maintaining the integrity of skip connections. The GoogLeNet architecture incorporated inception modules to capture multiscale features effectively. Auxiliary classifiers within GoogLeNet assist in stabilizing the training process and improving convergence speed, leading to superior classification accuracy.

In contrast to deep learning models, classical machine learning baselines do not require iterative training over epochs. Instead, feature extraction was performed once per image, followed by direct classifier fitting on the training set. Feature normalization was applied prior to training, and model evaluation was performed using the same validation and test sets as the deep learning experiments to maintain consistency.

### Performance evaluation

Performance metrics are crucial for assessing the effectiveness of deep learning classification models, providing quantitative insights into a model’s ability to accurately predict class labels. A fundamental tool for this assessment is the confusion matrix, which compares predicted labels with actual labels and consists of four components: true positive (TP), true negative (TN), false positive (FP), and false negative (FN). Based on the confusion matrix, performance metrics such as accuracy, precision, recall, and F1-score are computed to evaluate classification performance (Eq. 1–4). Accuracy reflects the proportion of correct predictions out of the total predictions made (Anderson, 2018; Hernandez-Guedes et al., 2022; Khalid, 2024). In addition, model performance is evaluated using training and testing accuracy and loss curves (Eq. 5). A fivefold stratified cross-validation strategy was employed on the training set to ensure robust performance assessment given the relatively limited dataset size. For each fold, models were trained on four subsets and evaluated on the remaining subset, and the final performance was reported as the mean accuracy ± standard deviation across all folds. This approach provides a reliable estimate of model stability and generalization.

Training and testing accuracy and loss curves provide insight into model convergence and generalization behavior. Divergence between training and testing performance typically indicates overfitting or unstable learning.

1 $$\begin{array}{c}Accuracy= \frac{TP+TN}{TP+TN+FP+FN}\end{array}$$

2 $$\begin{array}{c}Precision = \frac{TP}{TP+FP}\end{array}$$

3 $$\begin{array}{c}Recall = \frac{TP}{TP+FN}\end{array}$$

4 $$\begin{array}{c}F1-score=2\frac{precision*recall}{precision+recall}\end{array}$$

5 $$\begin{array}{c}cross-entropy loss= -\sum_{i=1}^{N}\sum_{c=1}^{C}{y}_{i,c}.\mathrm{log}\left({\widehat{y}}_{i,c}\right)\end{array}$$

## Results and discussion

### Nitrate classification

**Fig. 9:**
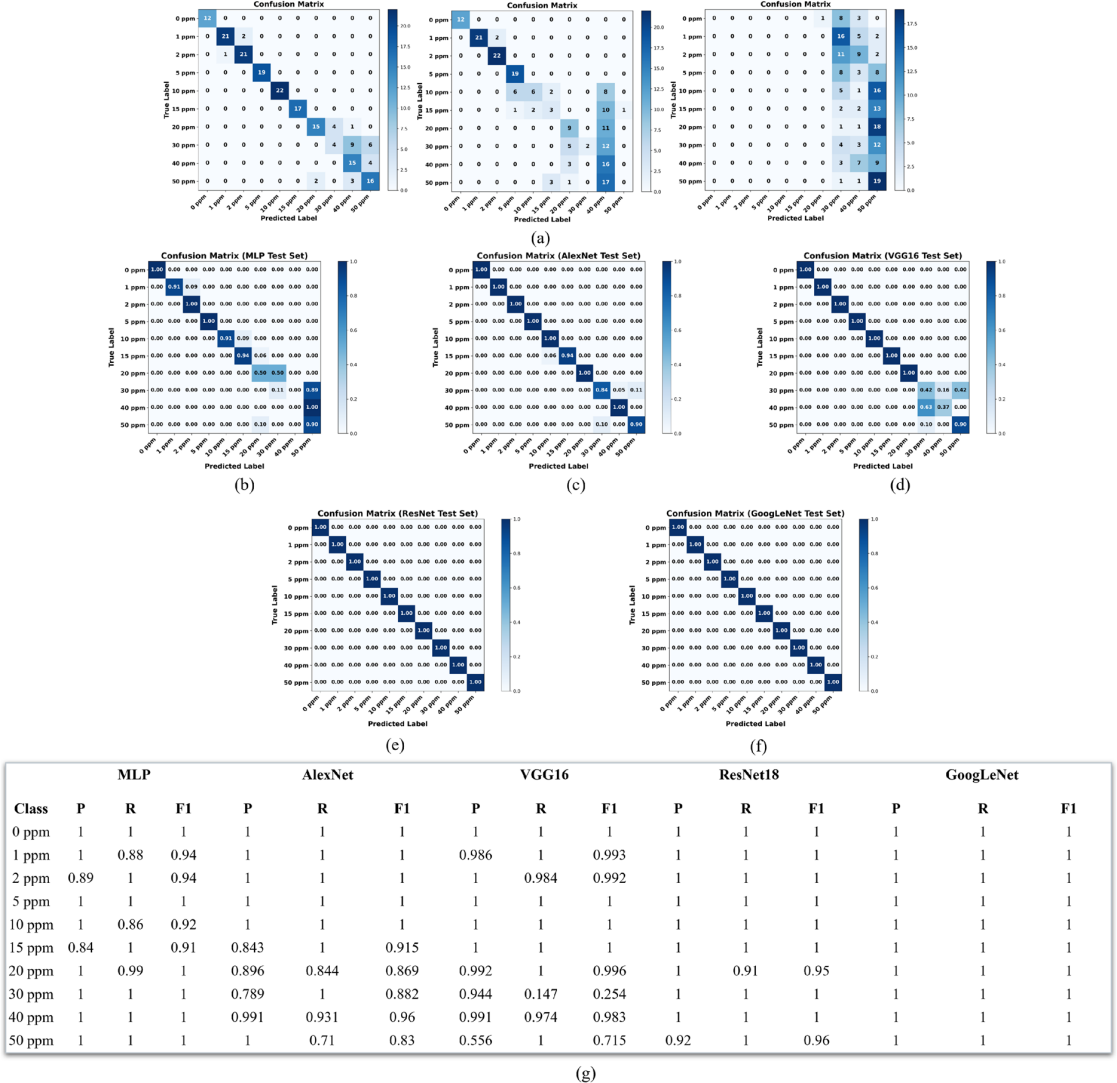
Confusion matrices for nitrate classification using different models: **a** classical baseline classifiers (logistic regression, linear support vector machine, and Naïve Bayes), **b** multilayer perceptron (MLP), **c** AlexNet, **d** VGG16, **e** ResNet18, and **f** GoogLeNet. Each confusion matrix shows the model’s ability to distinguish among ten nitrate concentration classes (0, 1, 2, 5, 10, 15, 20, 30, 40, and 50 ppm). Strong diagonal dominance indicates accurate classification, with ResNet18 and GoogLeNet demonstrating near-perfect performance across all concentrations. **g** Precision, recall, and F1-score values for each class and model

The performance of each model was evaluated on the independent test set using confusion matrices, along with accuracy, precision, recall, and F1-score metrics. These metrics provide a comprehensive assessment of both overall classification accuracy and class-wise behavior, highlighting patterns of misclassification and model robustness. The confusion matrices and detailed precision, recall, and F1-scores for nitrate classification are presented in Fig. 9.

Among the deep learning models, the MLP exhibited limited performance, particularly at higher nitrate concentrations. While lower concentration classes (0, 1, 2, 5, and 10 ppm) were classified reliably, substantial confusion occurred at 30, 40, and 50 ppm. Notably, the 40 ppm class was not correctly predicted at all, resulting in zero recall and F1-score, while the 30 ppm class achieved a recall of only 0.11 and an F1-score of 0.13. These results highlight the inherent limitation of MLP architectures for image-based tasks, as they lack spatial feature extraction capabilities.

AlexNet demonstrated a significant improvement over MLP, achieving an overall test accuracy of 96.9%. Most nitrate classes were classified with high precision and recall; however, moderate confusion was observed between adjacent concentrations, particularly at 30 ppm and 50 ppm, where F1-scores dropped to 0.86 and 0.90, respectively. Despite these errors, AlexNet showed strong generalization compared to simpler architectures.

VGG16 achieved a test accuracy of 89.2%, but exhibited inconsistent performance across mid-range nitrate concentrations. Although perfect classification was observed for several low and high concentration classes, the 30 ppm and 40 ppm classes suffered from notable misclassification, with F1-scores of 0.62 and 0.17, respectively. This variability may be attributed to VGG16’s large parameter count, which can lead to suboptimal generalization when training data are limited.

In contrast, ResNet18 and GoogLeNet achieved perfect classification performance, each reaching 100% test accuracy, with precision, recall, and F1-scores of 1.00 across all nitrate classes. The residual connections in ResNet18 and the multiscale feature extraction enabled by GoogLeNet’s inception modules contributed to stable optimization and exceptional generalization. Stratified fivefold cross-validation further confirmed this robustness, with mean accuracies exceeding 99.9% for both architectures.

In addition to deep learning models, classical machine learning baselines based on hand-crafted features were evaluated to contextualize the benefit of CNN-based approaches. The logistic regression baseline achieved a test accuracy of 83.5%, performing reasonably well at low concentrations but struggling to discriminate higher nitrate levels. The linear SVM model performed poorly, with a test accuracy of 56.7%, while the Naïve Bayes baseline failed to generalize effectively, achieving only 15.5% accuracy. These results demonstrate that color and texture features alone are insufficient to capture the subtle spatial variations present in colorimetric test strip images.

Overall, the results clearly indicate that modern CNN architectures, particularly ResNet18 and GoogLeNet, substantially outperform both simpler deep models and classical machine learning baselines, making them well suited for accurate and real-time nitrate concentration classification under standardized imaging conditions.

### Nitrite classification

**Fig. 10:**
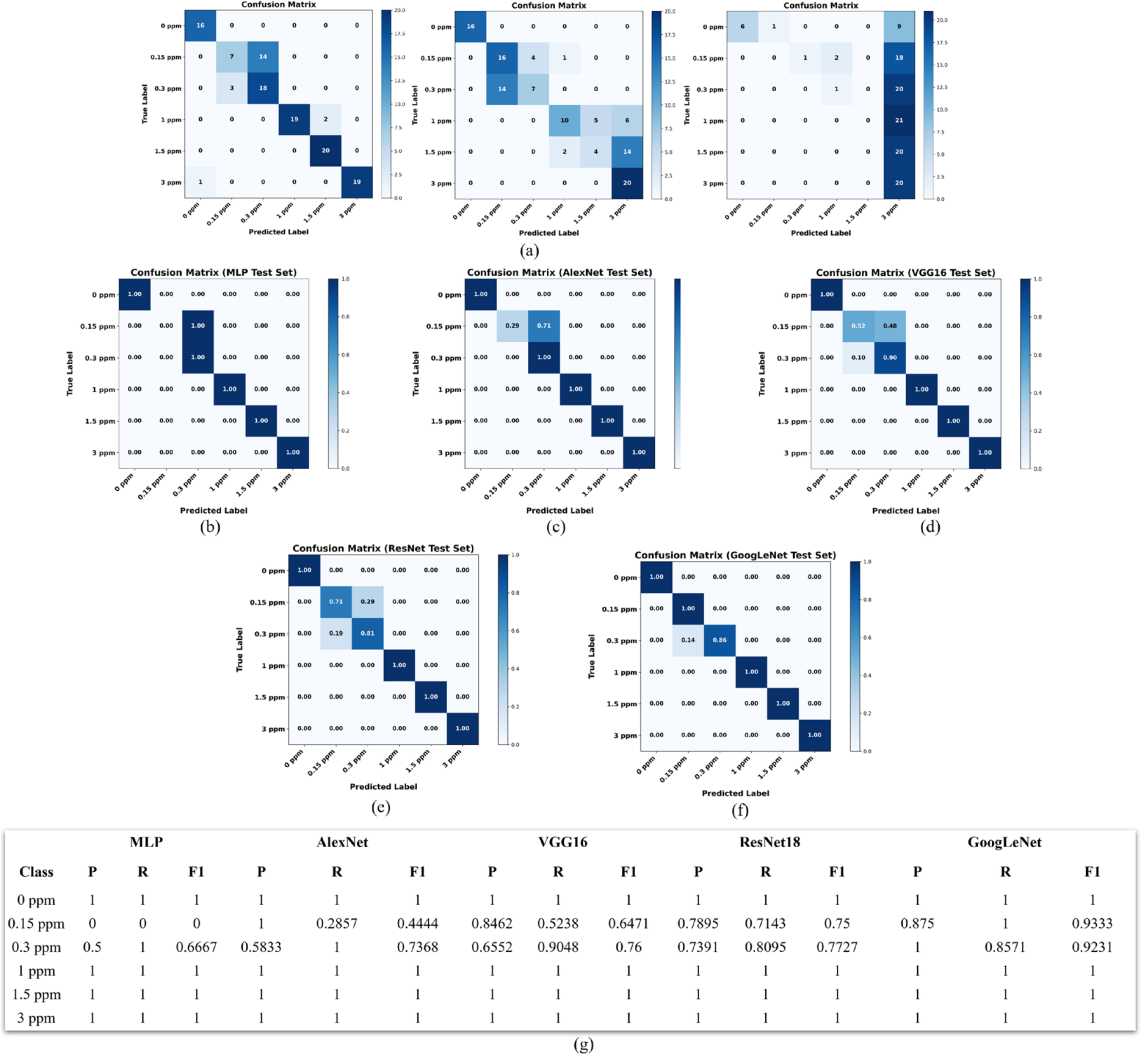
Confusion matrices for nitrite classification using different models: **a** classical baseline classifiers (logistic regression, linear support vector machine, and Naïve Bayes), **b** multilayer perceptron (MLP), **c** AlexNet, **d** VGG16, **e** ResNet18, and **f** GoogLeNet. Each confusion matrix shows the model’s ability to distinguish among six nitrite concentration classes (0, 0.15, 0.3, 1, 1.5, and 3 ppm). Strong diagonal dominance indicates accurate classification, with ResNet18 and GoogLeNet demonstrating near-perfect performance across all concentrations. **g** Precision, recall, and F1-score values for each class and model

The performance of the proposed models for nitrite classification was evaluated using confusion matrices and class-wise precision, recall, and F1-score metrics on the independent test set. The confusion matrices for nitrite classification are presented in Fig. 10, providing detailed insight into class-level prediction behavior and model robustness.

Among the deep learning models, GoogLeNet achieved the best overall performance, with a test accuracy of 97.5% and consistently high precision and recall across all nitrite concentrations. Minor misclassifications were observed only between the 0.15 ppm and 0.3 ppm classes, where subtle color variations and overlapping visual characteristics likely increased classification difficulty. Nevertheless, GoogLeNet maintained macro and weighted F1-scores above 0.97, demonstrating excellent generalization capability.

ResNet18 also showed strong performance, achieving a test accuracy of 91.6%. Most nitrite classes were classified reliably; however, moderate confusion occurred between the 0.15 ppm and 0.3 ppm classes, resulting in slightly reduced recall values (0.71 and 0.81, respectively). Despite these errors, ResNet18 maintained balanced performance across all concentrations, supported by a macro F1-score of 0.92.

In contrast, MLP, AlexNet, and VGG16 showed comparatively lower performance, particularly for low-concentration nitrite classes. The MLP model failed to correctly identify the 0.15 ppm class, resulting in zero recall and a reduced test accuracy of 82.35%, highlighting the limitations of fully connected architectures for spatially complex image data. AlexNet and VGG16 achieved test accuracies of 87.39% and 89.92%, respectively, but both models exhibited noticeable confusion between visually similar low-level concentrations, especially 0.15 ppm and 0.3 ppm.

To further contextualize these results, classical machine learning baseline models were also evaluated using hand-crafted visual features. Logistic regression achieved a test accuracy of 83.19%, demonstrating that color information alone provides a reasonable baseline for nitrite estimation. However, this approach struggled with mid-range concentrations, where overlapping color distributions reduced class separability. A linear SVM yielded a lower test accuracy of 61.34%, indicating insufficient discriminative power for complex multi-class separation. The Naïve Bayes model performed poorly, achieving a test accuracy of only 21.85%, confirming that texture features alone are not suitable for nitrite strip classification.

Overall, these findings clearly demonstrate that deep CNN architectures significantly outperform classical hand-crafted feature-based models for nitrite classification. The superior performance of GoogLeNet and ResNet18 highlights the importance of hierarchical feature learning for capturing subtle color and spatial variations, making these architectures more suitable for reliable, high-precision nitrite monitoring under controlled imaging conditions.

### Model training and validation performance

**Fig. 11:**
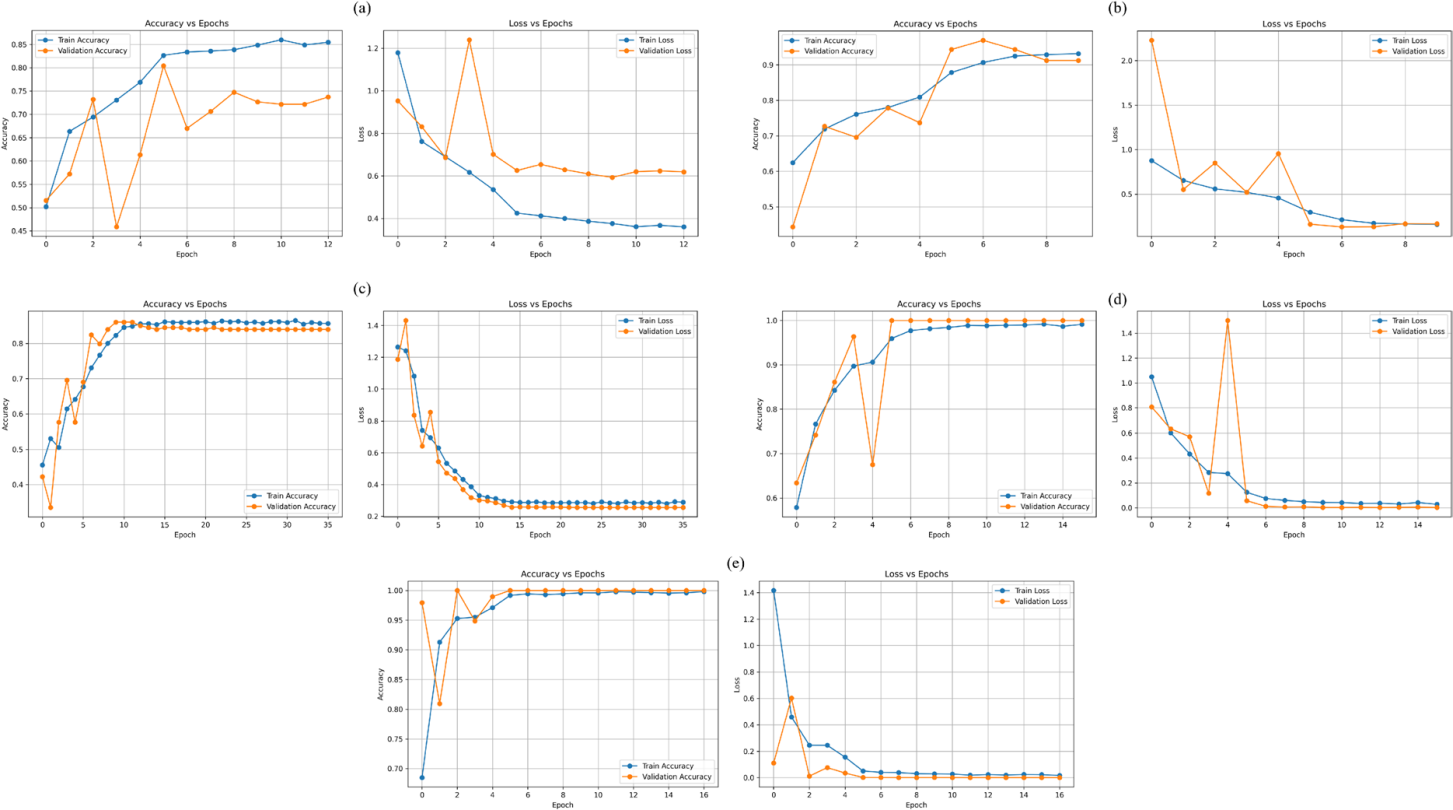
Training and testing accuracy and loss curves for nitrate classification using five deep learning models: MLP, AlexNet, VGG16, ResNet18, and GoogLeNet

**Fig. 12:**
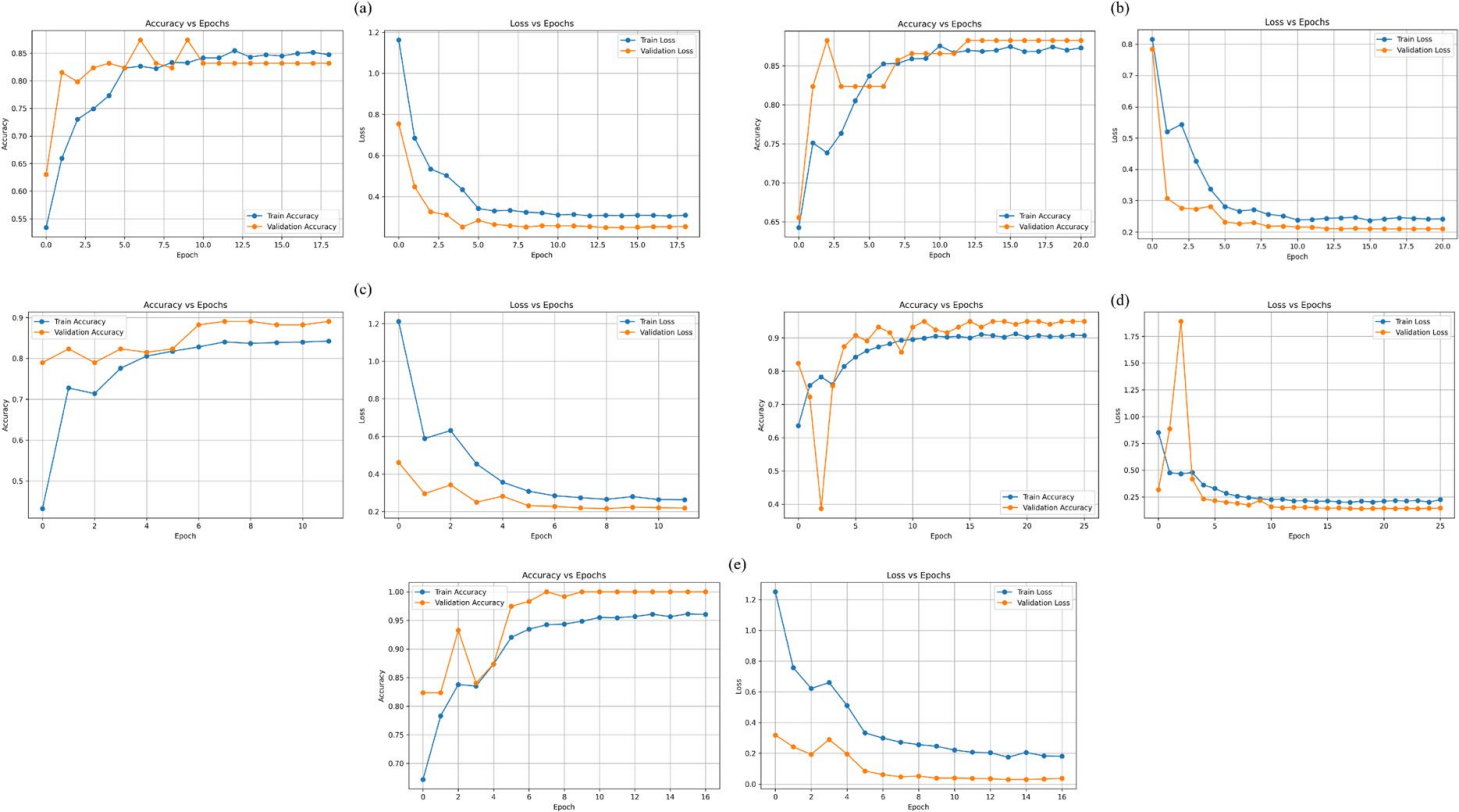
Training and testing accuracy and loss curves for nitrite classification using five deep learning models: MLP, AlexNet, VGG16, ResNet18, and GoogLeNet

The training and validation performance of the five deep learning models (MLP, AlexNet, VGG16, ResNet18, and GoogLeNet) for nitrate and nitrite classification was systematically analyzed using accuracy and loss curves, as shown in Figs. 11 and 12. These curves provide insight into convergence behavior, learning stability, and potential overfitting across epochs.

For nitrate classification (Fig. 11), all models exhibited a general increase in training accuracy accompanied by a decrease in training loss during the initial epochs, indicating effective feature learning. However, clear differences were observed in validation behavior. GoogLeNet and ResNet18 demonstrated highly stable validation accuracy curves that closely followed their training accuracy, along with consistently decreasing validation loss. This strong alignment between training and validation trends indicates excellent generalization and minimal overfitting.

AlexNet showed moderate fluctuations in validation accuracy and loss across epochs, suggesting sensitivity to intra-class variations among nitrate concentrations. VGG16 exhibited a slower convergence rate with higher validation loss in early epochs, but its performance gradually improved as training progressed, reflecting the impact of its deeper architecture on feature extraction. The MLP model showed the slowest convergence and the largest gap between training and validation accuracy, highlighting its limited ability to capture spatial and chromatic patterns from image data.

Overall, GoogLeNet and ResNet18 achieved the most consistent and reliable training–validation behavior for nitrate classification.

For nitrite classification (Fig. 12), all models showed an overall increase in training accuracy and a decrease in training loss across epochs, indicating effective learning. However, greater variability was observed in validation behavior compared to nitrate classification, reflecting the increased complexity of nitrite strip patterns. GoogLeNet demonstrated the most stable performance, with closely aligned training and validation accuracy curves and consistently decreasing validation loss, indicating strong generalization. ResNet18 also achieved high accuracy with relatively smooth convergence, despite minor early fluctuations in validation performance.

In contrast, VGG16 exhibited moderate instability during early epochs, with fluctuating validation accuracy and loss before stabilizing. AlexNet showed larger oscillations in validation accuracy and occasional validation loss spikes, suggesting sensitivity to data variability. The MLP displayed the slowest convergence and the largest gap between training and validation accuracy, highlighting its limited capacity to capture complex features. Overall, GoogLeNet and ResNet18 provided the most reliable performance for nitrite classification.

### Model comparison

Table 4 presents a comprehensive comparison of deep learning models and classical baseline approaches for both nitrate and nitrite classification. The comparison includes training, validation, and test accuracies, test loss, cross-validation performance, and computational efficiency, enabling a clear assessment of performance, generalization, and deployment feasibility across model families.

**Table 4:** Performance comparison of deep learning and classical baseline models

	Architecture	Train Acc	Val Acc	Test Acc	Test Loss	5-fold CV Acc (mean ± std)	Training time (s)	Inference time (ms/sample)
Nitrate	Logistic regression	0.8098	0.8247	0.8351	—	—	< 1	0.0039
	Linear SVM	0.5603	0.5361	0.5670	—	—	< 1	0.0037
	Naïve Bayes	0.3455	0.1649	0.1546	—	—	< 1	0.0072
	MLP	0.8545	0.7371	0.7268	0.5949	0.7392 ± 0.0484	75.44	0.0312
	AlexNet	0.9316	0.9124	0.9691	0.171	0.9437 ± 0.0068	99.3	0.1934
	VGG16	0.8571	0.8402	0.8711	0.3161	0.8484 ± 0.0343	652.22	0.5924
	ResNet18	0.9913	1	1	0.0022	0.9997 ± 0.0004	128.51	1.8655
	GoogLeNet	0.9979	1	1	0.0006	0.9997 ± 0.0004	187.93	0.843
Nitrite	Logistic Regression	0.8382	0.8487	0.8319	—	—	< 1	0.0079
	Linear SVM	0.5943	0.6555	0.6134	—	—	2.03	0.0055
	Naïve Bayes	0.4404	0.2185	0.2185	—	—	< 1	0.0089
	MLP	0.8474	0.8319	0.8235	0.2498	0.8346 ± 0.0117	69.23	0.0216
	AlexNet	0.8729	0.8824	0.8739	0.2074	0.8742 ± 0.0132	98.92	0.1172
	VGG16	0.8427	0.8908	0.8992	0.2170	0.8600 ± 0.0213	326.26	0.5547
	ResNet18	0.9070	0.9496	0.9160	0.1480	0.8868 ± 0.0275	122.25	0.2762
	GoogLeNet	0.9603	1	0.9748	0.0870	0.9522 ± 0.0117	141.67	0.4671

#### a) Deep learning models

For nitrate classification, deep learning models consistently outperformed classical baselines across all evaluation stages. ResNet18 and GoogLeNet demonstrated the strongest and most stable performance, achieving near-perfect training accuracies (0.9913 and 0.9979), validation accuracies of 1.0000, and test accuracies of 1.0000, with very low-test losses (0.0022 and 0.0006, respectively). The close alignment between training, validation, test, and cross-validation results (0.9997 ± 0.0004 for both models) indicates excellent generalization and minimal overfitting. These results highlight the effectiveness of deeper architectures with residual connections and inception modules in capturing subtle chromatic and spatial variations in nitrate test strip images.

Among the remaining deep models, AlexNet achieved high performance with a test accuracy of 0.9691, supported by strong training (0.9316) and validation (0.9124) accuracies, though minor confusion between adjacent concentration classes remained. VGG16 showed moderate performance (test accuracy 0.8711) with a noticeable reduction in cross-validation accuracy (0.8484 ± 0.0343) and substantially higher training time, suggesting increased sensitivity to harder or ambiguous samples. The MLP baseline exhibited the weakest deep learning performance, with a clear drop from training accuracy (0.8545) to test accuracy (0.7268), underscoring the limitations of fully connected architectures for image-based classification tasks.

For nitrite classification, a similar performance hierarchy was observed. GoogLeNet again demonstrated the strongest results, achieving training, validation, and test accuracies of 0.9603, 1.0000, and 0.9748, respectively, with a low-test loss (0.0870) and stable cross-validation performance (0.9522 ± 0.0117). ResNet18 also showed strong generalization (test accuracy, 0.9160; CV, 0.8868 ± 0.0275), though performance was slightly reduced compared to nitrate classification, reflecting the increased difficulty of distinguishing low nitrite concentration levels. AlexNet and VGG16 demonstrated moderate and consistent performance, while MLP again showed reduced robustness, particularly in the presence of subtle inter-class variations.

#### b) Classical baseline models

Classical baseline methods provided a useful reference for quantifying the benefit of deep feature learning. For nitrate classification, logistic regression (LR) achieved the strongest baseline performance, with training, validation, and test accuracies of 0.8098, 0.8247, and 0.8351, respectively. In contrast, linear SVM and Naïve Bayes showed substantially lower performance, with test accuracies of 0.5670 and 0.1546, indicating limited discriminative capability.

A similar trend was observed for nitrite classification, where logistic regression again produced the best baseline results (train, 0.8382; val, 0.8487; test, 0.8319). Linear SVM and Naïve Bayes failed to generalize effectively, particularly at lower concentration levels. Across both datasets, classical baselines exhibited larger discrepancies between training and test performance and higher inter-class confusion, highlighting their limited ability to model subtle color and texture variations compared to deep learning approaches.

#### c) Computational efficiency and practical implications

From a computational perspective, GoogLeNet and ResNet18 offered a favorable balance between accuracy and efficiency, achieving strong generalization with inference times below 1 ms per sample, making them suitable for real-time, on-site deployment. Although VGG16 achieved reasonable classification performance, its significantly higher training and inference costs reduced its practical appeal. Classical baseline models were computationally lightweight but did not achieve sufficient accuracy for reliable deployment.

Overall, the results in Table 4 demonstrate that modern deep convolutional architectures—particularly GoogLeNet and ResNet18—provide substantial and consistent performance gains over classical baseline classifiers (LR, SVM, NB). The strong agreement between training, validation, test, and cross-validation results supports their suitability for robust, automated, vision-based nitrate and nitrite monitoring systems under controlled imaging conditions.

### Evaluation using independent field samples

To further evaluate the practical applicability of the proposed system, independent real water samples were collected from field locations and analyzed in the laboratory using an AQ2 discrete analyzer at South Dakota State University (SDSU) to obtain reference nitrate and nitrite concentrations. The same samples were subsequently tested using the colorimetric strips and classified by the trained GoogLeNet model. As shown in Table 5, the predicted concentration classes generally aligned with the corresponding laboratory-measured values, with most samples correctly assigned to the nearest discrete concentration level. Minor deviations were primarily observed near class boundaries, as expected given the categorical nature of colorimetric strip readings and the inherent variability in low-concentration ranges. These results demonstrate that the proposed vision-based approach can provide reliable categorical estimates on real water samples under standardized imaging conditions, supporting its potential for rapid, on-site screening applications. Further evaluation under diverse environmental conditions is planned to assess robustness beyond controlled laboratory settings.

**Table 5:** AQ2 laboratory measurements and corresponding GoogLeNet predictions for nitrate and nitrite concentrations in real field water samples

Sample name	Actual nitrate ppm	GoogLeNet prediction ppm	Actual nitrite ppm	GoogLeNet prediction ppm
Sample 1 (B1)	5.2445	5	0.05275	0
Sample 2 (B2)	1.4935	2	0.0047	0
Sample 3 (AUTO NORTH)	5.0275	5	0.3257	0.3
Sample 4 (AUTO SOUTH)	11.281	10	0.02265	0
Sample 5 (B3)	3.4655	5	0.02675	0
Sample 6 (B4)	0.44	0	0.02005	0
Sample 7 (B1)	2.631	2	0.0063	0
Sample 8 (B2)	0.6665	0	0.05735	0
Sample 9 (AUTO NORTH)	3.077	5	0.0076	0
Sample 10 (B3)	2.1795	2	0.008	0
Sample 11 (B4)	0.183	0	0.03165	0
Sample 12 (B1)	1.0215	1	0.0062	0
Sample 13 (AUTO NORTH)	1.8695	2	0.0081	0
Sample 14 (AUTO SOUTH)	1.551	2	0.0062	0
Sample 15 (B3)	1.7325	2	0.0157	0
Sample 16 (B4)	0.287	0	0.0333	0

## Conclusion

This study presents a computer vision–based approach for classifying nitrate and nitrite concentrations using colorimetric test strip images. Multiple deep learning models, including MLP, VGG16, AlexNet, ResNet18, and GoogLeNet, were evaluated using the same datasets under consistent preprocessing and training conditions. Among these, ResNet18 and GoogLeNet consistently achieved the strongest performance, exhibiting near-perfect classification accuracy for nitrate and high accuracy (up to 97.48%) for nitrite classification under standardized imaging conditions. While models such as MLP, VGG16, and AlexNet served as comparative baselines, AlexNet notably underperformed due to its earlier-generation CNN architecture and limited capacity for multiscale feature representation, which hindered its ability to capture fine-grained spatial variations in the test strip images. Performance was generally lower for nitrite classification, likely due to greater intra-class variability, which demands stronger feature representation. Overall, the findings highlight the effectiveness of modern, deeper CNN architectures for developing accurate and reproducible vision-based systems for water quality monitoring under controlled imaging conditions. The current evaluation was conducted under controlled imaging conditions and does not explicitly assess robustness to environmental variations such as illumination changes, noise, strip orientation, or partial occlusion. The proposed solution shows strong potential for real-time processing and on-site deployment under standardized imaging conditions, particularly in resource-constrained settings. Future work will focus on optimizing and deploying the proposed models on low-power edge devices (e.g., NVIDIA Jetson–class platforms or Raspberry Pi–based systems) capable of real-time, on-device inference within an enclosed imaging setup, as well as evaluating robustness under broader environmental conditions to support practical and accessible water quality monitoring.

## Data Availability

Data will be provided on request.
